# Slower saccadic reading in Parkinson’s disease

**DOI:** 10.1371/journal.pone.0191005

**Published:** 2018-01-24

**Authors:** Naz Jehangir, Caroline Yizhu Yu, Jeehey Song, Mohammad Ali Shariati, Steven Binder, Jill Beyer, Veronica Santini, Kathleen Poston, Yaping Joyce Liao

**Affiliations:** 1 Department of Ophthalmology, Stanford University School of Medicine, Stanford, California, United States of America; 2 Department of Neurology, Stanford University School of Medicine, Stanford, California, United States of America; University of Toronto, CANADA

## Abstract

Idiopathic Parkinson’s Disease (PD) is characterized by degeneration of dopaminergic and other neurons, leading to motor and non-motor deficits. Abnormal eye movements in PD, including fixations, saccades, and convergence, are well described. However, saccadic reading, which requires serial and alternating saccades and fixations, is not well studied, despite its obvious impact on the quality of life. In this study, we assessed saccadic reading using variations of the King-Devick (KD) test, a rapid single digit number naming test, as a way to assess the ability to make serial left-to-right ocular motor movements necessary for reading. We recruited 42 treated PD patients and 80 age-matched controls and compared their reading times with a variety of measures, including age, duration of disease, Unified Parkinson’s Disease Rating Scale (UPDRS), the National Eye Institute 25-Item Visual Functioning Questionnaire 25 (VFQ-25), and Montreal Cognitive assessment (MoCA) test. The subjects performed 4 trials of reading 120 single digit numbers aloud as fast as possible without making errors. In each trial, they read 3 pages (KD1, KD2, and KD3), and each page contained 40 numbers per page in 8 lines with 5 numbers/line. We found that PD patients read about 20% slower than controls on all tests (KD1, 2, and 3 tests) (p < 0.02), and both groups read irregularly spaced numbers slower than regularly spaced numbers. Having lines between numbers to guide reading (KD1 tests) did not impact reading time in both PD and controls, but increased visual crowding as a result of decreased spacing between numbers (KD3 tests) was associated with significantly slower reading times in both PD and control groups. Our study revealed that saccadic reading is slower in PD, but controls and PD patients are both impacted by visuospatial planning challenges posed by increased visual crowding and irregularity of number spacing. Reading time did not correlate with UPDRS or MoCA scores in PD patients but significantly correlated with age, duration of disease, and VFQ-25 scores. The presence of convergence insufficiency did not significantly correlate with reading time in PD patients, although on average there was slower reading time in those with convergence insufficiency by 8 s (p = 0.2613). We propose that a simple reading task using 120 single-digit numbers can be used as a screening tool in the clinical setting to assess functional ocular motor difficulties in Parkinson’s disease that can have a profound impact on quality of life.

## Introduction

Parkinson’s disease (PD) is the second most common neurodegenerative condition after Alzheimer’s disease and has a prevalence of 425 per 100,000 from ages 64–75 years [1–3]. PD is characterized by progressive motor (bradykinesia, tremors, rigidity and gait problems) and non-motor symptoms, including cognitive impairment, dementia, depression, insomnia, and autonomic dysfunction [4–7]. Other than loss of dopaminergic neurons and their connections in the substantia nigra and basal ganglia that are well described in PD patients [8], studies have also reported atrophy in different brain areas in PD patients, including areas important for memory formation and cognitive function [9, 10]. Various factors including fatigue, depression, slowness, tremors and writing difficulties may result in impairment of activities of daily living and employment issues in PD patients [11].

Although not broadly recognized, PD patients often have visual symptoms due to ophthalmic and neurologic issues, including blurred vision, double vision, visual hallucinations, and difficulty reading, impacting their quality of life [12–16]. Ophthalmic issues that are common in PD include refractive errors, dry eye (related to reduced blink), meibomian gland disease, and increased rate of primary open angle glaucoma [13]. Retinal issues can present as impaired contrast sensitivity, difficulty with light adaptation, color vision deficits, and abnormal electroretinogram, which may be related to loss of retinal dopaminergic neurons [16, 17]. Optical coherence tomography (OCT) is a useful non-invasive tool that has been used to measure retinal structural changes in PD patients, showing variable amount of thinning from neurodegeneration in various studies [17–22].

Other than afferent visual issues, PD patients likely suffer from visual disability due to eye movement issues, including hypometric saccades, fixation instability from saccadic intrusions, convergence insufficiency, and other ocular motor difficulties [12, 14, 23–26]. Convergence insufficiency is common in PD patients and can result in double vision and difficulty with reading and other near work related issues [27]. PD patients have also been reported to have visuospatial and cognitive issues that can impact ocular motor planning, visual behavior, and reading comprehension [9, 28–31]. The King-Devick (KD) test is a rapid number naming test that has been used to assess number reading in PD patients [14], concussion [32–34], fatigue such as sleep deprivation [35], and neurological disorders such as multiple sclerosis [36] and Alzheimer’s disease [37, 38]. In this study, we used variations of the KD single digit rapid number naming test using both irregular and regular spacing, in order to assess ocular motor and visuospatial abilities during left-to-right reading in the absence of semantic context.

## Materials and methods

### Patient recruitment

All experimental procedures were approved by Stanford Institutional Review Board and consistent with the Declaration of Helskinki. Informed, written consent was obtained from all subjects following detailed verbal discussion on the purpose of the study and how the tasks are performed. We measured number reading speeds in 42 PD patients (30 males, 12 females, mean age: 68.3 ± 1.7 years, range: 40–84 years) and 80 controls (31 males, 49 females, mean age: 66 ± 1.1 years, range: 50–87 years). Mean disease duration in PD patients was 7.9 ± 0.9 years (range: 0.17–21 years). All 42 PD patients were on dopaminergic treatment per movement disorders specialist (100% on medical treatment alone and 24% with deep brain stimulation and medical treatment). To measure the health related quality of life, the National Eye Institute 25-Item Visual function Questionnaire (VFQ-25) was administered in 35/42 PD patients and 77/80 controls at the same time as they were being tested for the reading task. Of the 42 PD subjects, 27 (64%) had the Unified Parkinson’s Disease Rating Scale (UPDRS) scores (mean 27.85 ± 2.06), and all patients were on treatment. The Montreal Cognitive assessment (MoCA) test score was available in 22 (52.4%) patients (mean 25.82 ± 0.72). Patients with probable idiopathic Parkinson’s disease (no autopsy data available to confirm clinically definite PD) were recruited from the Byers Eye Institute neuro-ophthalmology clinic at Stanford University. Inclusion criteria for PD patients included the diagnoses of idiopathic Parkinson’s disease by a movement disorders specialist, currently on appropriate treatment for PD, having good enough vision, eye movement control, and cognitive abilities to participate in the study, and absence of ophthalmic and other neurological issues that may impact vision or reading. Controls were recruited from the same institution in the optometry and general ophthalmology clinic, and we felt this population, which had vision complaints but no uncorrectable ophthalmic or neurological diseases was a reasonable control group for PD patients who presented to the eye clinic for evaluation in the same location. All subjects had comprehensive ophthalmic measurements and were permitted to wear their own corrective lenses for reading.

### Number reading assessments

We used variations of the King-Devick rapid number reading test to assess ocular motor behavior necessary in reading [14, 39]. All PD subjected were on treatment for PD at the time of testing. The reading task was explained to the subjects, and they were informed that their reading times would be recorded. The subjects were shown a demonstration page while being instructed on how to read. The person administering the test provided reassurance throughout testing to minimize any anxiety and tracked any errors or technical issues. Subjects were given opportunity to practice if desired and to re-start if errors were made. All subjects read the same tests in the same order.

**Fig 1:**
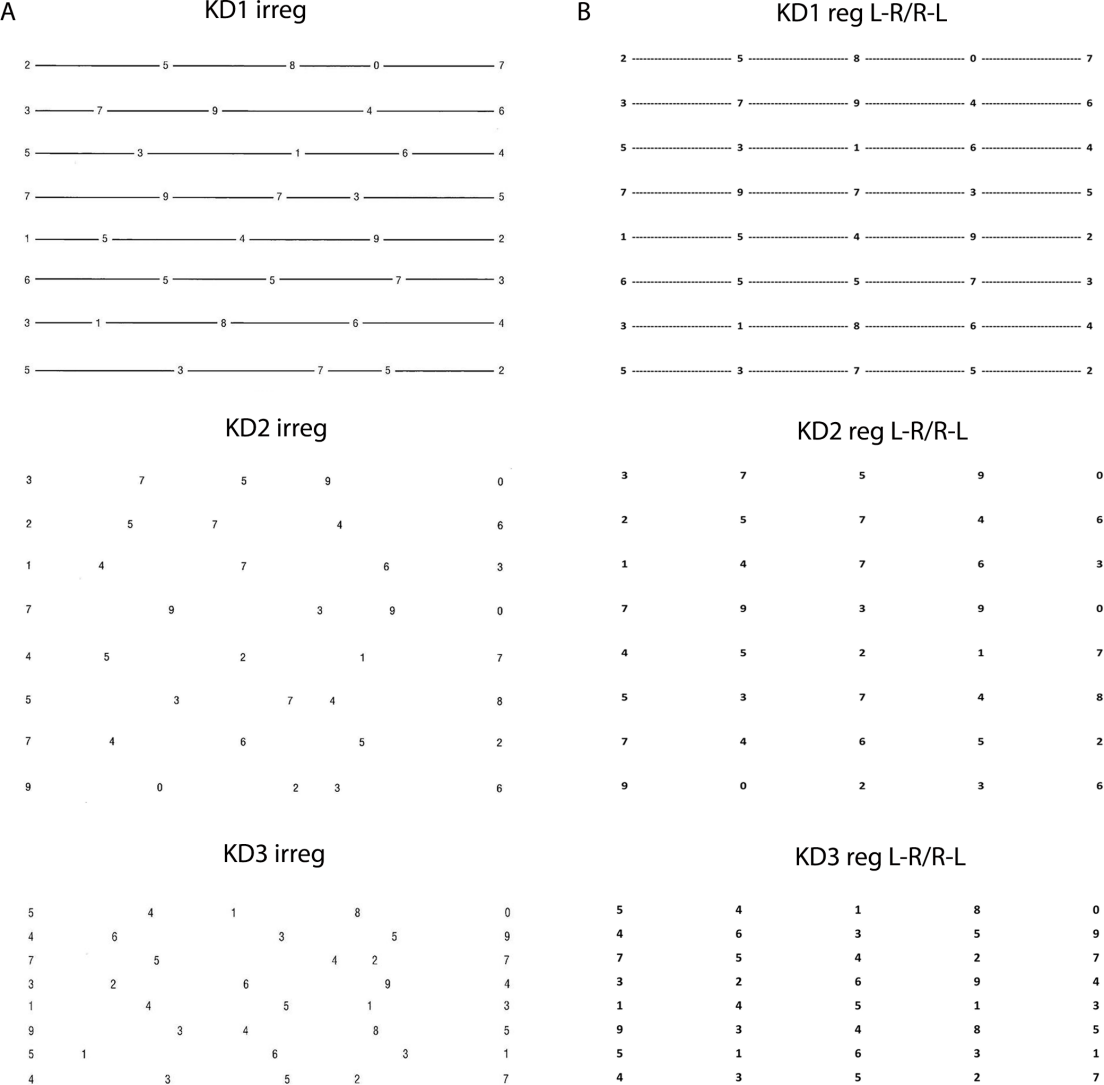
**Irregularly (A) and regularly (B) spaced single digit number reading tests.** The irregularly spaced tests (KD1 irreg, KD2 irreg, and KD3 irreg) were read left-to-right twice. Then the regularly spaced tests (KD1 reg, KD2 reg, and KD3 reg) were read left-to-right (L-R) and then right-to-left (R-L). KD1 has a horizontal line to help guide reading from one number to the next, although that adds some visual crowding. KD2 has the same spacing as KD1 but has no lines to guide reading. KD3 has less vertical space between the lines compared with KD2 and therefore has the most amount of visual crowding.

The subjects performed 4 trials of reading 120 single digit numbers aloud as fast as possible without making errors. In each trial, they read 3 pages (KD1, KD2, and KD3), and each page contained 40 numbers per page in 8 lines with 5 numbers/line (Fig 1). In the first 2 trials, the subjects read the irregularly spaced numbers (KD123 irreg) from left-to-right as fast as possible, and the faster of the 2 times was used for the study. In the 3^rd^ trial, the subjects read regularly spaced numbers from left-to-right (KD123 reg L-R), and in the 4^th^ trial, the subjects read the regularly spaced numbers from right-to-left (KD123 reg R-L). No repetition for the regular tasks was done in order to minimize fatigue. Repetition was also not needed because the subjects already knew how to perform the test. The 3 irregular reading pages (KD123 irreg) were the same as the King-Devick tests [12, 14]. For the regularly spaced tests (KD123 reg), we recreated the KD test with the same numbers, x- and y-dimensions, size of each number except the numbers were evenly spaced.

### Data analysis

Statistical analysis was performed using non-parametric tests. We used the Mann-Whitney U test to perform intergroup analysis (PD and controls) and the Wilcoxon signed rank test for intragroup analysis. Linear regression model was used to assess correlation among different measurements. The level of significance was set at p < 0.05.

## Results

### Significantly slower reading in PD compared with age-matched controls

**Fig 2:**
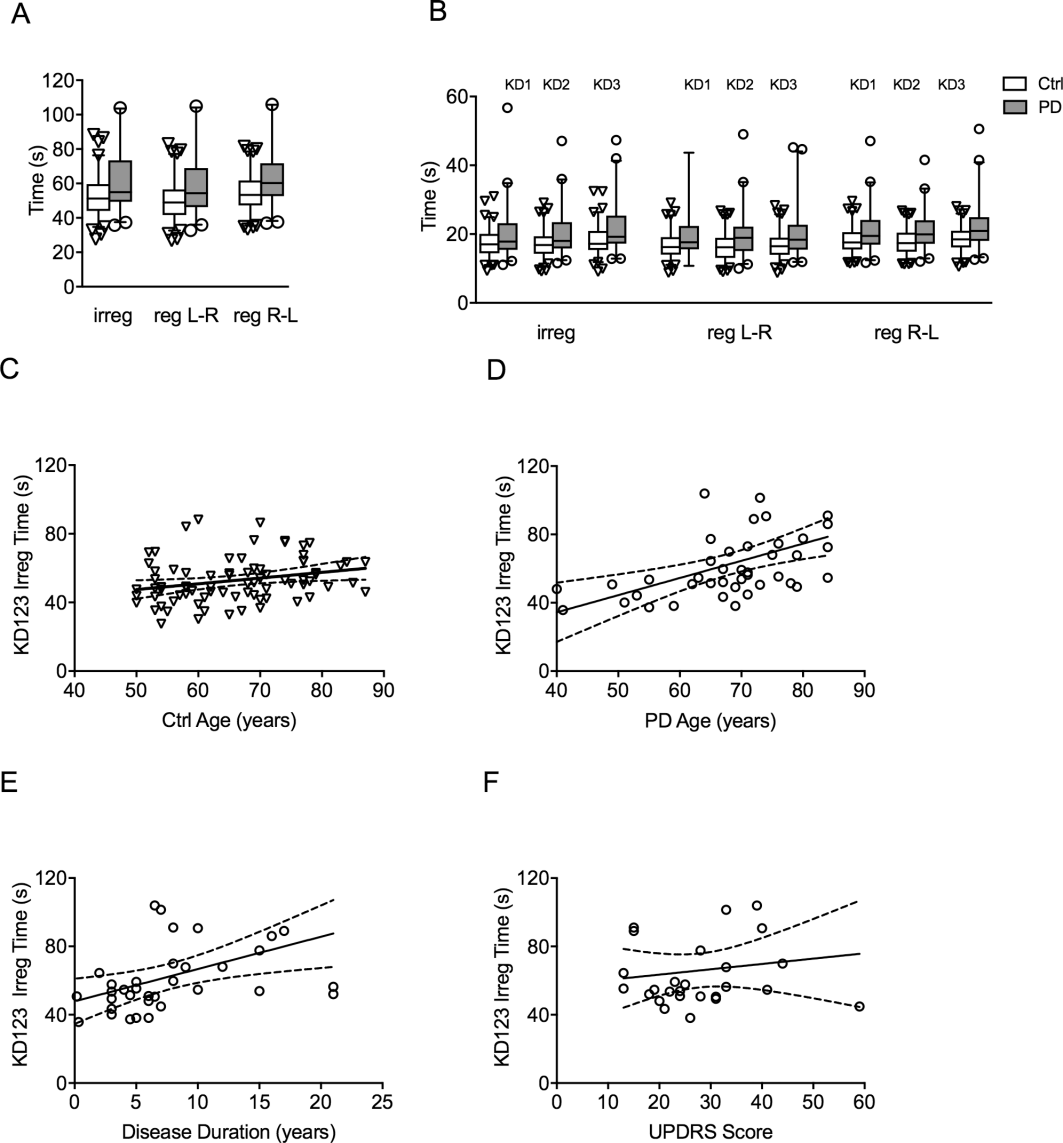
PD patients read significantly slower in all reading tasks. **(A)** Box-and -whisker plot of sum of KD1, KD2 and KD3 (KD123) reading times (3 pages or 120 numbers per task) for KD123 irreg, KD123 reg L-R, and KD123 reg R-L. **(B)** Box-and-whisker plot of individual KD reading times (1 page, 40 numbers per task). (**C**) Significant correlation between age and KD123 irreg reading time in the control group (R^2^ = 0.06853 p = 0.0206). (**D**) Significant correlation between age and KD123 irreg reading times in PD (R^2^ = 0.02389, p = 0.0010). (**E**) Significant correlation between disease duration and KD123 irreg reading time in PD group (R^2^ = 0.1905 p = 0.0088). (**F**) No correlation between UPDRS score and KD123 irreg reading time (R^2^ = 0.01811 p = 0.5034).

We compared reading times in 80 controls and 42 treated PD patients using 120 single digit numbers that were 1) irregularly spaced and read left-to-right (KD123 irreg), 2) regularly spaced and read left-to-right (KD123 reg L-R, and 3) regularly spaced and read right-to-left (KD123 reg R-L) (Fig 1, Methods). In the KD123 irreg task (3 pages of 40 single digit numbers, irregularly spaced, read left-to-right), the PD patients read 10 s or 19% slower compared with controls (ctrl: 52.9 ± 1.4 s or 2.27 numbers/s, PD: 62.8 ± 3.5 s or 1.91 numbers/s; p = 0.0155, Mann-Whitney) (Table 1, Fig 2). Using the regularly spaced task KD123 reg L-R, the PD patients read 10 s or 21% slower compared with controls (ctrl: 49.9 ± 1.3 s, PD: 60.2 ± 3.3 s; p = 0.0105, Mann-Whitney). Comparing with reading from left-to-right (KD123 irreg and KD123 reg L-R), reading from right-to-left (KD123 reg R-L), a direction that is less practiced in English readers, both controls and the PD groups read significantly slower (KD123 irreg vs. KD123 reg R-L: ctrl p < 0.0001, PD p = 0.0183; KD123 reg L-R vs. KD123 reg R-L: ctrl p< 0.0001, PD p< 0.0001). In addition, the PD patients read 11 s or 20% slower compared with controls (ctrl: 54.4 ± 1.3 s, PD: 65.2 ± 3.1 s; p = 0.0023, Mann-Whitney) (Table 1, Fig 2A and Fig 2B).

**Table 1:** Reading time in different number reading tests.

	Control	Parkinson’s Disease pts	Ctrl vs PD pts p-values
Reading Tests	Time (s) N = 80	Time (s) N = 42	(Mann-Whitney)
**KD123 irreg**	52.9 ± 1.4	62.8 ± 3.5	0.0155
**KD123 reg L-R**	49.9 ± 1.3	60.2 ± 3.3	0.0105
	(p < 0.0001*)	(p = 0.0045*)	
**KD123 reg R-L**	54.4 ± 1.3	65.2 ± 3.1	0.0023
	(p < 0.0001*)	(p = 0.018*)	
**KD1 irreg**	17.4 ± 0.5	20.3 ± 1.3	0.0658
**KD1 reg L-R**	16.6 ± 0.4	19.5 ± 1.0	0.0178
	(p < 0.0001*)	(p = 0.2*)	
**KD1 reg R-L**	18.2 ± 0.5	21.4 ± 1.1	0.0080
	(p = 0.0002*)	(p = 0.0028*)	
**KD2 irreg**	17.2 ± 0.5	20.6 ± 1.2	0.0203
**KD2 reg L-R**	16.5 ± 0.46	19.9 ± 1.1	0.0076
	(p = 0.0003*)	(p = 0.9761*)	
**KD2 reg R-L**	17.7 ± 0.4	21.3 ± 1.0	0.0019
	(p = 0.00046*)	(p = 0.0160*)	
**KD3 irreg**	18.2 ± 0.5	21.9 ± 1.2	0.0093
**KD3 reg L-R**	16.76 ± 0.4	20.7 ± 1.3	0.0088
	(p < 0.0001*)	(p = 0.0067*)	
**KD3 reg R-L**	18.5 ± 0.4	22.5 ± 1.2	0.0025
	(p = 0.0047*)	(p = 0.2627*)	

KD123 irreg is sum of reading time for irregularly spaced KD1, KD2, and KD3 test cards read from left-to-right. KD123 reg L-R is sum of KD1, KD2 and KD3 test cards with regularly spaced numbers and read from left-to-right. KD123 reg R-L is sum of KD1, KD2 and KD3 reg tests, read from right-to-left instead of left-to-right direction.

*Abbreviations*: K-D, King Devick; irreg, irregular; reg, regular; L-R, left to right; R-L, right to left; PD pts, Parkinson’s disease patients; Ctrl, control; s, seconds.

*p values calculated using Wilcoxon signed-rank test.

There was significant correlation between reading time and age for PD and control groups in the KD123 irregular reading task (ctrl, R^2^ = 0.0685 p = 0.0206; PD, R^2^ = 0.02389 p = 0.0010) (Fig 2C and Fig 2D) and in the KD123 L-R regular reading task (ctrl, R^2^ = 0.09155, p = 0.0071; PD R^2^ = 0.1631, p = 0.0080). Both KD123 irreg and reg reading times significantly correlated with disease duration in the PD group (KD123 irreg: R^2^ = 0.1905, p = 0.0088; KD123 reg L-R: R^2^ = 0.1872, p = 0.0094) (Fig 2E) but not with UPDRS score (KD123 irreg: R^2^ = 0.01811, p = 0.5034; KD123 reg L-R: R^2^ = 0.02122, p = 0.4685) (Fig 2F) or MoCA score (KD123 irreg: R^2^ = 0.0075, p = 0.7021; KD123 reg L-R: R^2^ = 0.0097, p = 0.6624).

We compared the reading time with the VFQ-25 scores in both PD patients and controls. In the PD group, the mean composite VFQ-25 score was significantly worse in compared with the controls (ctrl 89.2 ± 0.72 s; PD 77.3 ± 1.82 s, p < 0.0001, Mann-Whitney test) and significantly correlated with reading time (KD123 irreg: R^2^ = 0.1372, p = 0.0285; KD123 reg L-R: R^2^ = 0.1171, p = 0.0472). In the control group, the VFQ-25 score was not significantly correlated with reading time (KD123 irreg: R^2^ = 0.00013, p = 0.9218; KD123 reg L-R: R^2^ = 0.00307, p = 0.6322). In the control group, the 20 slowest readers had VFQ25 mean score of 89 ± 1 which was the same as the 20 fastest control readers mean score of 90 ± 1 (p = 0.6918, Mann-Whitney test).

In the PD group, 53% had convergence insufficiency, and 47% had normal convergence at near. There was slower reading time in those with convergence insufficiency (mean 62.5 ± 4.6 s, range 44.3–104.0 s) compared with those without (mean 71 ± 6.5 s, range 43.5–151.0 s), but reading time did not significantly correlate with convergence insufficiency (p = 0.2613, Mann-Whitney test).

### PD patient read slower when numbers are irregularly spaced

Looking at the individual pages of reading (e.g. KD1, KD2, KD3), the PD patients read significantly slower in every task by 2–4 seconds or 16–21% (Table 1, Fig 2B).

In controls, the KD123 irreg reading time was slower by 3.0 s compared with KD123 reg L-R reading time (KD123 irreg: 52.9 ± 1.4 s, KD123 reg: 49.9 ± 1.3 s, p < 0.0001). In the PD group, the total irregular reading time was slower by 2.7 s compared with total regular reading time (KD123 irreg: 62.8 ± 3.5 s, KD123 reg L-R: 60.2 ± 3.3 s, p = 0.000452). In the controls, the individual time (KD1, KD2, KD3) was significantly faster in each of the regular tasks compared with the irregular tasks (e.g. KD1 irreg vs. KD1 reg) (p < 0.005). In the PD group, only the individual time for the KD3 task was significantly faster (KD3 irreg vs. KD3 reg, p = 0.00672) but not the KD1 or the KD2 times, because the patients read slower both in the irreg and the reg tasks.

### The effect of visual crowding in reading

We compared reading time in the individual KD1, 2, and 3 pages to assess the effect of visual crowding. KD2 has the least amount of visual crowding, then KD1 (extra horizontal lines between numbers), and KD3 has the most amount of visual crowding (reduced space between lines of reading) (Fig 1). Comparing KD2 vs. KD1 reading times by the *same* subjects, there was no significant difference in the control and PD groups (KD1 vs. KD2 in irreg, reg L-R, or reg R-L tasks; Table 1). In the control group, KD1 irreg and KD2 irreg reading times were 17.4 s and 17.2 s. In the PD group, KD1 irreg and KD2 irreg times were 20.3 s and 20.6 s respectively. Comparing KD2 vs. KD3 reading times by the same subjects, the control and PD groups consistently read slower. The control group read KD3 slower compared with KD2 by 0.3–1 s in KD3 (irreg: p < 0.0001; reg L-R: p = 0.03752, reg R-L: p < 0.0001). The PD patients read KD3 slower compared with KD2 by 0.8–1.3 s (irreg: p = 0.006, reg L-R: p = 0.6818, reg R-L: p = 0.0466) (Table 1).

## Discussion

Reading difficulty is common in PD and several factors contribute to this difficulty including ocular motor abnormalities, visuospatial difficulties, and cognitive issues. In this study, we used variations of single digit number reading tests to examine the ocular motor abilities necessary in effective reading in 42 PD and 80 control subjects in the absence of semantic context. Similar to previously published studies [12, 14], we found that the reading time for irregularly spaced single digit numbers (King-Devick test) was significantly slower in PD compared with controls (KD123 sum). PD patients also read slower when reading regularly spaced numbers (KD123 irreg vs. KD123 reg). Increased visual crowding by reducing the space between each line of reading (KD3) was the slowest for both controls and PD groups, suggesting that both groups were similarly affected by visual crowding.

Slower reading of single digit numbers can be attributed to a number of reasons, including eye movement abnormalities. Ocular motor pattern required in saccadic reading consists of making rapid, alternating fixations and small saccades from left-to-right across a line and then efficiently return to the next line [40], which is affected in PD [41]. Impairment of saccades and smooth pursuit eye movements has been reported in about 75% of PD patients [42]. Some factors contributing to slow reading in these patients include longer duration of fixations and a reduced ability to generate saccades. Execution of eye movement involves the frontal eye fields, supplemental eye field and the parietal eye fields. Functional MRIs (magnetic resonance imaging) in PD patients have demonstrated underactivity in the frontal eye fields and the supplemental eye fields and a relative hyperactivity in the parietal fields [43, 44].

Slower number reading can also be due to visuospatial planning difficulties, which are affected in PD [9, 29–31]. In a study of 76 PD patients and 76 age-matched controls where the subjects are asked to assess the inclination of each pair of lines on a page (Benton’s judgment of line orientation test), PD patients exhibit significantly greater horizontal line errors and complex intra-quadrant errors compared with controls [45]. Visuospatial deficits correlate with age, level of education, disease duration and severity [45]. In another study, neuropsychological testing to assess cognition shows that in early stage PD patients (disease duration less than 2 years), there is no deficit in attention, verbal abilities and select visuospatial skills. However, the same patients exhibit significant cognitive issues such as verbal memory, figural material recalling, and some visuospatial issues such as facial recognition [31].

Visual crowding also contributes to slower reading. Visual crowding is a phenomenon that results in impaired recognition of a target secondary to presence of other objects in the peripheral visual field, making it difficult to identify the target [46]. Crowding contributes to the slow reading seen in dyslexics and amblyopes. Single letters are easily identified than words in both groups owing to less crowding with single letters [47]. Other than visuospatial deficits, PD patients also have grammatical impairment and perform worse with sentences containing complex clauses than simple clausal sentences [48, 49]. Lee used a simpler approach using a word detection technique (minimizing task demand) in PD patients without clauses and observed that these patients have limited sensitivity to phonetic errors in a grammatical morpheme compared to controls [29]. Our data showed that visual crowding by having lines between numbers did not significantly increase reading time in PD. Also, although the lines between the numbers can theoretically facilitate reading from one number to the next, this was not the case. Further increase in visual crowding by decreased spacing between numbers affected the reading speed in both PD and control groups in a similar way.

Although extremely important as a part of daily living, reading abilities are not typically assessed in the clinical setting. A number reading test can be a useful measure since it is rapidly done in the clinical setting. The KD test is one such test that can be readily used to assess ocular motor abilities necessary in reading that is relatively not impacted by the native language abilities. Prolong KD reading time has been well documented in PD patients [12, 14]. Moster and colleagues observed prolongation of total KD time in a group of 81 patients with multiple sclerosis by 13.5 seconds (average difference) compared to 20 age matched controls groups [36]. Similarly, in our study we observed PD patients read slower by 10–11 seconds (KD123 sum) compared with controls. KD was originally developed to assess reading skills in pre-school children [50] and can be readily used to test those who speak different languages. Dodick et al. also show in a randomized study that repeated training with the King-Devick Reading Acceleration Program Software in first and second grade children leads to improved fluency and comprehension as assessed by the Wechsler Individual Achievement Test [51].

Slow reading time has been correlated with several factors. Lin observed no significant correlation between reading time and UPDRS score or the duration of disease [14]. In our study reading time did not significantly correlate with UPDRS and MOCA scores but we observed a significant correlation with age and duration of disease. We observed a higher mean VFQ-25 score in controls than PD patients and a correlation was seen between the KD123 reading times and the VFQ-25 scores only in the PD group. PD patients have been noted to have a worse vision related quality of life (measured by VFQ-25 questionnaires) compared with controls [15] and this can severely impact affect their overall quality of life by compromising their activities of daily living [27].

There are several limitations of this study. We measured reading using single digit numbers rather than words in the absence of semantic context, which is not the same as reading words[52, 53]. This was done in order to assess the ocular motor abilities necessary for reading using the easiest test possible, while minimizing the effects of educational level and higher order cognitive functions. We have unpublished data that showed a significant correlation between reading of single digit numbers aloud vs. silent reading of words in paragraphs, using infrared oculography to ensure that all subjects did not skip lines (p < 0.0001). Another important limitation to the study is the confounding effects of reading aloud, which can be more difficult in some PD patients, although this would impact all the trials similarly for any one subject. A reading test that is done aloud is easier in the clinical setting when infrared oculography is not available, in order to provide immediate feedback that the task is performed correctly. By studying PD patients on treatment, we tried to reduce the difficulty of speaking to a minimum. Reading aloud, silently, or both have been used in different reading studies in patients with vision issues [54–58] with silent reading being more affected in some studies[55, 56] but not in all and a significant correlation between reading aloud and silent reading has been also observed [58] Comparison of silent and reading aloud has not been done in PD patients. Moreover no single standardized test exists for evaluation of silent reading [55, 59]. Although we did not control for prior reading abilities and educational background, we assumed that they had minimal impact on number reading because numbers are introduced very early in education and are prevalent in daily living. We did not assess for other behavioral problems such as anxiety and did not perform specific behavioral measures because KD test is considered to be an easy and low stress test. Finally, there may be a selection bias since both control and PD subjects were recruited from an eye clinic, but this should have affected the two groups similarly.
